# Activation of Water‐Splitting Photocatalysts by Loading with Ultrafine Rh–Cr Mixed‐Oxide Cocatalyst Nanoparticles

**DOI:** 10.1002/anie.201916681

**Published:** 2020-03-06

**Authors:** Wataru Kurashige, Yutaro Mori, Shuhei Ozaki, Masanobu Kawachi, Sakiat Hossain, Tokuhisa Kawawaki, Cameron J. Shearer, Akihide Iwase, Gregory F. Metha, Seiji Yamazoe, Akihiko Kudo, Yuichi Negishi

**Affiliations:** ^1^ Department of Applied Chemistry Faculty of Science Tokyo University of Science, 1–3 Kagurazaka, Shinjuku-ku Tokyo 162-8601 Japan; ^2^ Research Institute for Science & Technology Tokyo University of Science, 1–3 Kagurazaka, Shinjuku-ku Tokyo 162-8601 Japan; ^3^ Department of Chemistry University of Adelaide Adelaide South Australia 5005 Australia; ^4^ Department of Chemistry Graduate School of Science Tokyo Metropolitan University 1-1 Minami-Osawa, Hachioji-shi Tokyo 192-0397 Japan

**Keywords:** cocatalysts, metal clusters, nanostructures, photocatalysts, water splitting

## Abstract

The activity of many water‐splitting photocatalysts could be improved by the use of Rh^III^–Cr^III^ mixed oxide (Rh_2−*x*_Cr_*x*_O_3_) particles as cocatalysts. Although further improvement of water‐splitting activity could be achieved if the size of the Rh_2−*x*_Cr_*x*_O_3_ particles was decreased further, it is difficult to load ultrafine (<2 nm) Rh_2−*x*_Cr_*x*_O_3_ particles onto a photocatalyst by using conventional loading methods. In this study, a new loading method was successfully established and was used to load Rh_2−*x*_Cr_*x*_O_3_ particles with a size of approximately 1.3 nm and a narrow size distribution onto a BaLa_4_Ti_4_O_15_ photocatalyst. The obtained photocatalyst exhibited an apparent quantum yield of 16 %, which is the highest achieved for BaLa_4_Ti_4_O_15_ to date. Thus, the developed loading technique of Rh_2−*x*_Cr_*x*_O_3_ particles is extremely effective at improving the activity of the water‐splitting photocatalyst BaLa_4_Ti_4_O_15_. This method is expected to be extended to other advanced water‐splitting photocatalysts to achieve higher quantum yields.

## Graphical abstract

**Rh_2−*x*_** **Cr**_***x***_ **O_3_ particles** with a size of approximately 1.3 nm were loaded onto a BaLa_4_Ti_4_O_15_ photocatalyst. The photocatalyst exhibited the highest quantum yield reported for BaLa_4_Ti_4_O_15_. The developed preparation technique of Rh_2−*x*_Cr_*x*_O_3_ particles is also expected to improve the activity of other advanced water‐splitting photocatalysts. SG=glutathionate.

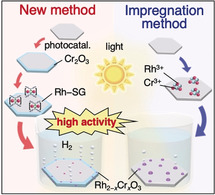

## Introduction

With increasing the global warming and the depletion of fossil resources, society is expected to shift to using clean and renewable energy instead of fossil fuels. Hydrogen (H_2_) can be converted to electricity by using a fuel cell and such conversion generates only water as a by‐product. Therefore, the establishment of a system in which H_2_ is generated from water and solar energy by using a photocatalyst^1^ is desirable, with the generated H_2_ used for the generation of electricity from fuel cells (Scheme S1). Once such an energy‐conversion system is established, it will be possible to circulate an energy medium (H_2_) in addition to obtaining electricity only from solar energy and abundant water resources. However, realization of such an ultimate energy‐conversion system requires further improvement of the reaction efficiency of water splitting as well as fuel cells.

**Scheme 1:**
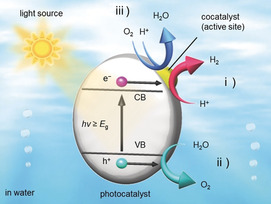
Schematic of photocatalytic water splitting using a one‐step photoexcitation system (CB, conduction band; VB, valence band; *E*_g_, band gap) showing the processes of i) hydrogen evolution, ii) oxygen evolution, and iii) oxygen photoreduction.

In many cases, the surface of water‐splitting photocatalysts is coated with metal or metal oxide nanoparticles.^2^, ^3^, ^4^, ^5^, ^6^, ^7^, ^8^, ^9^, ^10^, ^11^, ^12^, ^13^, ^14^, ^15^, ^16^, ^17^, ^18^, ^19^, ^20^, ^21^, ^22^ The loaded nanoparticles, called cocatalysts, promote charge separation and act as active sites in the water‐splitting reaction (Scheme 1). Effective strategies to realize highly active photocatalysts include improvement of the semiconductor photocatalyst and the cocatalyst. The activity of the cocatalysts can be enhanced by decreasing the particle diameter and improving the dispersibility.^2^, ^3^, ^4^, ^5^, ^6^, ^7^, ^8^, ^9^, ^10^, ^11^, ^12^, ^13^, ^14^, ^15^, ^16^, ^17^, ^18^, ^19^, ^20^, ^21^, ^22^ Previous studies have shown that the particle size of cocatalysts can be readily controlled in the small particle region when pre‐synthesized nanoparticles/clusters are used as precursors.^26^, ^27^, ^28^, ^29^, ^30^ In addition, previous studies have demonstrated that protection of the cocatalyst with a chromium oxide (Cr_2_O_3_) film prevents the photoreduction reaction of oxygen (O_2_) (Scheme 1), which is one of the back reactions of water splitting.^31^, ^32^, ^33^, ^34^, ^35^, ^36^, ^37^, ^38^, ^39^, ^40^, ^41^ We previously prepared a water‐splitting photocatalyst with relatively high activity by applying both of the aforementioned techniques to a gold (Au) cocatalyst‐supported BaLa_4_Ti_4_O_15_ photocatalyst (Figure S1).^42^, ^43^

However, a volcano plot of H_2_ adsorption and desorption^44^ predicted that rhodium (Rh) should show higher activity than Au as a cocatalyst for H_2_ production. Therefore, it is expected that loading an ultrafine cocatalyst composed of Rh and Cr oxides onto a photocatalyst surface will lead to higher water‐splitting activity. Actually, Maeda and co‐workers^45^, ^46^, ^47^, ^48^ reported that a photocatalyst loaded with Rh^III^–Cr^III^ mixed oxide nanoparticles (Rh_2−*x*_Cr_*x*_O_3_, 10–30 nm) showed higher water‐splitting activity than a photocatalyst loaded with other nanoparticles. The activity of the photocatalyst seemed to increase with the use of finer Rh_2−*x*_Cr_*x*_O_3_ nanoparticles. However, it is very difficult to load ultrafine (<2 nm) particles onto photocatalysts by using common methods, such as impregnation or photodeposition.^45^, ^46^, ^47^, ^48^ In addition, it is also difficult to load the small Rh_2−*x*_Cr_*x*_O_3_ particles onto the photocatalysts by using the loading method developed in the previous studies^42^, ^43^ because there are no precise synthesis method for small Rh_2−*x*_Cr_*x*_O_3_ clusters, which is different from the case with Au clusters.

In this study, we attempted to load small Rh_2−*x*_Cr_*x*_O_3_ cluster cocatalysts, by using the aggregation of a Rh–thiolate complex, onto the surface of the photocatalysts. As a result, we have succeeded in loading Rh_2−*x*_Cr_*x*_O_3_ particles with a size of approximately 1.3 nm and a narrow size distribution onto a BaLa_4_Ti_4_O_15_ photocatalyst (Rh_2−*x*_Cr_*x*_O_3_(1.3 nm)/BaLa_4_Ti_4_O_15_). Scanning transmission electron microscopy coupled with energy‐dispersive X‐ray spectroscopy (STEM‐EDX) and X‐ray absorption fine structure (XAFS) measurements revealed that the loaded particles were an oxide solid solution composed of Rh^III^ and Cr^III^ species. Moreover, Rh_2−*x*_Cr_*x*_O_3_(1.3 nm)/BaLa_4_Ti_4_O_15_ showed higher water‐splitting activity than that of previously reported BaLa_4_Ti_4_O_15_ loaded with fine (≈1.2 nm) Au‐cocatalyst nanoparticles protected by a Cr_2_O_3_ film^42^, ^43^ and BaLa_4_Ti_4_O_15_ loaded with ≈3 nm Rh_2−*x*_Cr_*x*_O_3_ particles (Rh_2−*x*_Cr_*x*_O_3_(3.0 nm)/BaLa_4_Ti_4_O_15_) prepared via the impregnation method.

## Results and Discussion

**Figure 1:**
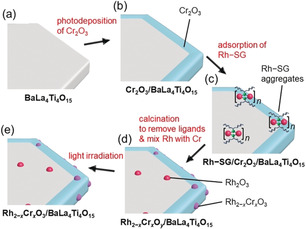
Schematic of the experimental procedure. (a) BaLa_4_Ti_4_O_15_, (b) Cr_2_O_3_/BaLa_4_Ti_4_O_15_, (c) Rh–SG/Cr_2_O_3_/BaLa_4_Ti_4_O_15_, (d) Rh_2−*x*_Cr_*x*_O_*y*_/BaLa_4_Ti_4_O_15_, and (e) Rh_2−*x*_Cr_*x*_O_3_/BaLa_4_Ti_4_O_15_. Rh_2−*x*_Cr_*x*_O_*y*_ indicates Rh_2−*x*_Cr_*x*_O_3_ including highly oxidized Cr (>3+).

BaLa_4_Ti_4_O_15_ (Figure S1), which is a most advanced photocatalyst, was used in this work. Rh_2−*x*_Cr_*x*_O_3_ particles (≈1.3 nm) were loaded onto the BaLa_4_Ti_4_O_15_ surface by the method summarized in Figure 1. Firstly, a Cr_2_O_3_ layer was formed on BaLa_4_Ti_4_O_15_ (Figure 1 a) by photodeposition to form Cr_2_O_3_/BaLa_4_Ti_4_O_15_ (Figure 1 b).^42^, ^43^ Then, a Rh–glutathionate (SG, Figure S2) complex with a molecular size slightly under 1 nm was adsorbed on the surface of Cr_2_O_3_/BaLa_4_Ti_4_O_15_ to give Rh–SG/Cr_2_O_3_/BaLa_4_Ti_4_O_15_ (Figure 1 c). Next, Rh–SG/Cr_2_O_3_/BaLa_4_Ti_4_O_15_ was calcined at 300 °C under reduced pressure to remove the ligands from the Rh–SG complex and form a solid solution of Rh and Cr oxides (Rh_2−*x*_Cr_*x*_O_*y*_/BaLa_4_Ti_4_O_15_, Figure 1 d). Finally, a small amount of Cr with a high oxidation state (*y*>3) was reduced to Cr^III^ by using ultraviolet (UV) light irradiation (Rh_2−*x*_Cr_*x*_O_3_(1.3 nm)/BaLa_4_Ti_4_O_15_, Figure 1 e).^42^, ^43^

**Figure 2:**
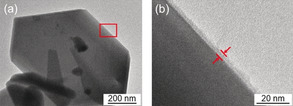
HR‐TEM images of Cr_2_O_3_/BaLa_4_Ti_4_O_15_ observed at (a) low and (b) high magnification for the edge of BaLa_4_Ti_4_O_15_ (Figure S3 and S4). The image (b) is an expansion of the red square in image (a). In this experiment, Cr was loaded at 1 wt % to easily monitor the position of the Cr_2_O_3_ layers. For the HR‐TEM image of Cr_2_O_3_/BaLa_4_Ti_4_O_15_ with 0.1 wt %, see Figure S5.

The high‐resolution (HR)‐TEM images of the Cr_2_O_3_/BaLa_4_Ti_4_O_15_ is shown in Figure 2. The Cr_2_O_3_ layers were mainly observed at the edge of BaLa_4_Ti_4_O_15_ (Figure S3–S5). The reduction of the metal ions occurs more easily at the edge of BaLa_4_Ti_4_O_15_ than at the flat surface of BaLa_4_Ti_4_O_15_,^49^ therefore, the Cr_2_O_3_ layers seem to be formed preferentially at the edge of BaLa_4_Ti_4_O_15_ (Figure 1 a).

**Figure 3:**
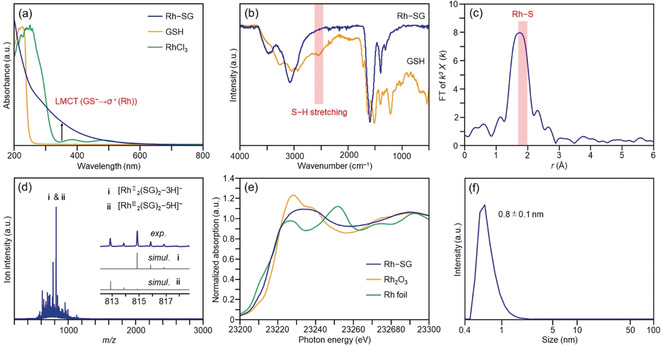
Characterization of the Rh–SG complex. (a) Comparison of the optical absorption spectra of Rh–SG, RhCl_3_, and GSH. (b) Comparison of FT‐IR spectra of Rh–SG and GSH (Figure S6). (c) Rh K‐edge FT‐EXAFS of Rh–SG. (d) Negative‐ion ESI mass spectrum of Rh–SG (Figure S7), showing that the main products are Rh_2_(SG)_2_ containing Rh^II^ (i) or Rh^III^ (ii) (Table S1). (e) Comparison of K‐edge XANES spectra of Rh–SG, Rh_2_O_3_, and Rh foil, confirming that Rh in Rh–SG is oxidized. (f) DLS spectrum of Rh–SG, showing that the Rh–SG complex has an average diameter of 0.8 nm in water.

The Rh–SG complex was prepared by mixing RhCl_3_ with glutathione (GSH, Figure S2) in water, followed by the addition of a reducing agent. The optical absorption spectrum of the product is shown in Figure 3 a. A peak attributed to charge transfer from SG to Rh (ligand‐to‐metal charge transfer; LMCT)^50^ was observed near 350 nm, indicating the formation of the Rh−S bond. This bond formation was also confirmed by Fourier transform (FT) infrared (IR) absorption^51^ (Figure 3 b and S6) and Rh K‐edge FT extended X‐ray absorption fine structure (EXAFS) spectroscopies^52^ (Figure 3 c). The electrospray ionization (ESI) mass spectrum of the product (Figure S7) is shown in Figure 3 d. This spectrum revealed that the Rh–SG complex mainly contained Rh^II^ and Rh^III^ species (Figure 3 d and Table S1). A similar interpretation was also obtained from the Rh K‐edge X‐ray absorption near‐edge structure (XANES) spectrum (Figure 3 e); the XANES spectrum is not consistent with that of Rh^III^ because of a distribution in the charge state of Rh in Rh–SG complex. A dynamic light scattering (DLS) measurement revealed that the obtained Rh–SG complex had a molecular size of around 0.8 nm in solution (Figure 3 f).

The Rh–SG complex was adsorbed onto Cr_2_O_3_/BaLa_4_Ti_4_O_15_ by stirring the materials together in water (Figure 1 c). Inductively coupled plasma mass spectrometry (ICP‐MS) analysis of the supernatant confirmed that the Rh–SG complex was adsorbed onto the photocatalyst surface with a relatively high adsorption rate (Table S2). In water, the surface of Cr_2_O_3_ is in the state of CrO_(1.5−*m*)_(OH)_2*m*_⋅*x* H_2_O (*m=*0, 0.5, or 1.5).^53^ Moreover, the bare surface of BaLa_4_Ti_4_O_15_ lacking Cr_2_O_3_ has also hydroxyl groups (‐OH).^54^ It is considered that because the polar functional groups in the ligand of the Rh–SG complex, such as carboxyl and amino groups (Figure S2), formed hydrogen bonds with the ‐OH groups on the photocatalyst surface, the Rh–SG complex was adsorbed on Cr_2_O_3_/BaLa_4_Ti_4_O_15_ at a high adsorption rate (Figure S8 and S9).

**Figure 4:**
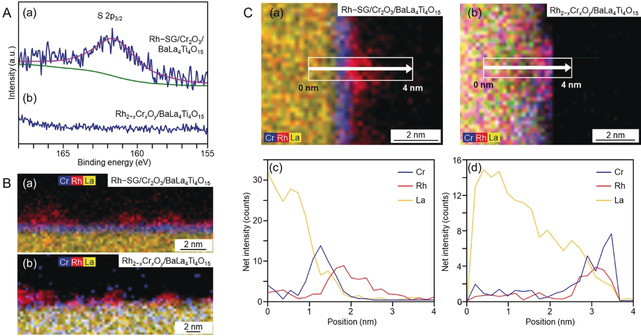
Characterization of the BaLa_4_Ti_4_O_15_ photocatalyst containing 0.09 wt % Rh and 0.10 wt % Cr. (A) S 2p photoelectron spectra and (B) STEM‐EDX elemental mapping for (a) Rh–SG/Cr_2_O_3_/BaLa_4_Ti_4_O_15_ and (b) Rh_2−*x*_Cr_*x*_O_*y*_/BaLa_4_Ti_4_O_15_. (C) Line analysis of elemental mapping for (a),(c) Rh–SG/Cr_2_O_3_/BaLa_4_Ti_4_O_15_ and (b),(d) Rh_2−*x*_Cr_*x*_O_*y*_/BaLa_4_Ti_4_O_15_.

**Figure 5:**
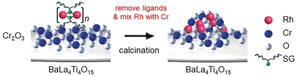
Schematic of the phenomenon that occurred during calcination process (Figure 4 B,C).

**Figure 6:**
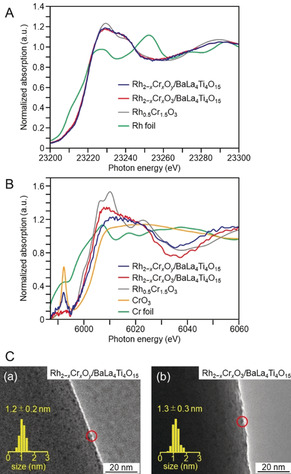
Characterization of the BaLa_4_Ti_4_O_15_ photocatalyst containing 0.09 wt % Rh and 0.10 wt % Cr. (A) Rh K‐edge XANES spectra of Rh_2−*x*_Cr_*x*_O_*y*_/BaLa_4_Ti_4_O_15_ and Rh_2−*x*_Cr_*x*_O_3_/BaLa_4_Ti_4_O_15_ together with those of Rh foil and Rh_0.5_Cr_1.5_O_3_. (B) Cr K‐edge XANES spectra of Rh_2−*x*_Cr_*x*_O_*y*_/BaLa_4_Ti_4_O_15_ and Rh_2−*x*_Cr_*x*_O_3_/BaLa_4_Ti_4_O_15_ together with those of Cr foil, CrO_3_, and Rh_0.5_Cr_1.5_O_3_. (C) TEM images of (a) Rh_2−*x*_Cr_*x*_O_*y*_/BaLa_4_Ti_4_O_15_ and (b) Rh_2−*x*_Cr_*x*_O_3_/BaLa_4_Ti_4_O_15_. In (C), the red circles indicate the Rh_2−*x*_Cr_*x*_O_*y*_ or Rh_2−*x*_Cr_*x*_O_3_ particles.

Rh–SG/Cr_2_O_3_/BaLa_4_Ti_4_O_15_ was calcined at 300 °C (Figure S10 and S11) under reduced pressure (>1.0×10^−1^ Pa; Figure 1 d). The S 2p photoelectron spectra before and after calcination of the BaLa_4_Ti_4_O_15_ photocatalyst containing 0.09 wt % Rh and 0.10 wt % Cr are shown in Figure 4 Aa and 4 Ab. Before calcination (Rh–SG/Cr_2_O_3_/BaLa_4_Ti_4_O_15_), a peak attributed to S 2p_3/2_ was observed near 161.2 eV (Figure 4 Aa).^55^ After calcination (Rh_2−*x*_Cr_*x*_O_*y*_/BaLa_4_Ti_4_O_15_), this peak disappeared (Figure 4 Ab), implying that SG was removed by calcination.^42^, ^43^ The elemental mapping of this photocatalyst before and after calcination is depicted in Figure 4 Ba and 4 Bb, respectively, obtained by STEM‐EDX. Before calcination (Rh–SG/Cr_2_O_3_/BaLa_4_Ti_4_O_15_), Rh was present on the Cr_2_O_3_ film (Figure 4Ba). After calcination (Rh_2−*x*_Cr_*x*_O_*y*_/BaLa_4_Ti_4_O_15_), both Rh and Cr were found in the same layer (Figure 4 Bb). This phenomenon was also confirmed from the line analysis of the elemental mapping (Figure 4 C). These results indicate that Rh and Cr species mixed during calcination (Figure 5). The Rh K‐edge XANES spectra of the photocatalyst before and after irradiation is shown in Figure 6 A. The Rh K‐edge XANES spectrum of the calcined sample (Rh_2−*x*_Cr_*x*_O_*y*_/BaLa_4_Ti_4_O_15_) greatly differs from that of Rh foil, but it resembles that of Rh_1.5_Cr_0.5_O_3_.^45^, ^46^, ^47^, ^48^ The estimated number of Rh−O bonds in the sample after calcination (Figure S12) is similar to that in Rh_2_O_3_ and Rh_1.5_Cr_0.5_O_3_ (Table S3). The Cr K‐edge XANES spectra of the sample before and after irradiation is depicted in Figure 6 B. After calcination (Rh_2−*x*_Cr_*x*_O_*y*_/BaLa_4_Ti_4_O_15_), the intensity of the Cr K‐edge XANES spectrum at an absorption edge near 6010 eV is stronger than that of Cr foil. This indicates that the Cr in the sample after calcination (Rh_2−*x*_Cr_*x*_O_*y*_/BaLa_4_Ti_4_O_15_) was oxidized.^56^ In the TEM image of Rh_2−*x*_Cr_*x*_O_*y*_/BaLa_4_Ti_4_O_15_ (Figure 6Ca), very fine particles (1.2±0.2 nm) were observed. These results indicate that calcination resulted in the loading of ultrafine Rh^III^–(Cr^III^, Cr^VI^) mixed oxide particles onto the BaLa_4_Ti_4_O_15_ surface. It is considered that because Rh and Cr tend to form mixed oxides when heated,^45^, ^46^ the calcination process induced the formation of mixed oxide particles as well as SG ligand removal (Figure 5).

Immediately after calcination (Figure 1 d), part of the Cr species in the sample was oxidized to a higher oxidation state than Cr^III^.^42^, ^43^ Then, the obtained photocatalyst was irradiated with UV light for 1 h to reduce the Cr species in the higher oxidation state to Cr^III^ (Figure 1 e).^42^, ^43^ The Cr K‐edge XANES spectrum confirmed that the Cr species in the higher oxidation state was reduced by the UV light irradiation (Figure 6 B and S13). This process had little effect on the oxidation state of the Rh species (Figure 6 A). Moreover, during the series of reaction steps in Figure 1, the crystalline structure of BaLa_4_Ti_4_O_15_ barely changed (Figure S14). In the TEM image of the obtained Rh_2−*x*_Cr_*x*_O_3_/BaLa_4_Ti_4_O_15_ (Figure 6 Cb), particles with a size of 1.3±0.3 nm were observed. These results demonstrate that the developed loading method enabled very fine Rh_2−*x*_Cr_*x*_O_3_ particles (≈1.3 nm) with a narrow size distribution to be loaded onto the BaLa_4_Ti_4_O_15_ surface (Rh_2−*x*_Cr_*x*_O_3_(1.3 nm)/BaLa_4_Ti_4_O_15_).

Considering the particle size of Rh_2−*x*_Cr_*x*_O_3_ of 1.3±0.3 nm, the obtained Rh_2−*x*_Cr_*x*_O_3_ particles should be formed from several Rh–SG complexes. Particles of this size were probably formed (Figure S15) because several Rh–SG complexes aggregated onto BaLa_4_Ti_4_O_15_ during the adsorption process (Figure 4 Ba, 4 Ca, and Figure 5). The size of the loaded Rh_2−*x*_Cr_*x*_O_3_ particles did not change when the loading amount of Rh was altered in the range of 0.05 to 0.13 wt % (Figure S16). This finding means that such a small difference of the concentration of Rh–SG complexes in solution hardly affected the degree of aggregation of Rh–SG complexes onto the BaLa_4_Ti_4_O_15_ surface during the adsorption process.

**Figure 7:**
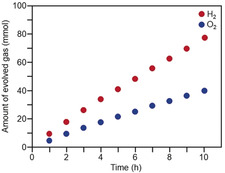
Time course of water splitting over Rh_2−*x*_Cr_*x*_O_3_(1.3 nm)/BaLa_4_Ti_4_O_15_ with 0.09 wt % Rh and 0.10 wt % Cr. The activity hardly decreased during 10 h of the water‐splitting reaction.

**Figure 8:**
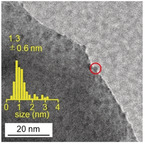
TEM image of Rh_2−*x*_Cr_*x*_O_3_(1.3 nm)/BaLa_4_Ti_4_O_15_ containing 0.09 wt % Rh and 0.10 wt % Cr after 10 h of the water‐splitting reaction. The average size of Rh_2−*x*_Cr_*x*_O_3_ particles after 10 h of the reaction was similar to that before the reaction (Figure 6 C(b)).

The water‐splitting activity of the obtained Rh_2−*x*_Cr_*x*_O_3_(1.3 nm)/BaLa_4_Ti_4_O_15_ photocatalyst was then examined. Specifically, Rh_2−*x*_Cr_*x*_O_3_(1.3 nm)/BaLa_4_Ti_4_O_15_ (500 mg) was dispersed in water and irradiated with UV light from a high‐pressure mercury (Hg) lamp (Figure S17).^42^, ^43^ Water‐splitting experiments using a series of samples (Figure S16 b) revealed that Rh_2−*x*_Cr_*x*_O_3_(1.3 nm)/BaLa_4_Ti_4_O_15_ containing 0.09 wt % Rh and 0.10 wt % Cr exhibited the highest activity (Figure S18). For this photocatalyst, almost no decrease in activity (Figure 7) and no increase in particle size (Figure 8) were observed even after 10 h of water‐splitting reaction. Moreover, it was confirmed that the reverse reaction (Figure S19 a and S20) and the O_2_ photoreduction reaction (Figure S19 b and S21) were well suppressed over this sample, as expected.

**Figure 9:**
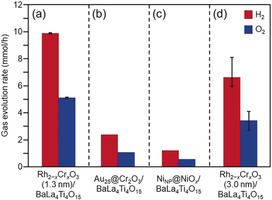
Comparison of gas‐evolution rates over different photocatalysts (a) Rh_2−*x*_Cr_*x*_O_3_(1.3 nm)/BaLa_4_Ti_4_O_15_ (0.09 wt % Rh and 0.10 wt % Cr), (b) Au_25_@Cr_2_O_3_/BaLa_4_Ti_4_O_15_ (0.10 wt % Au and 0.50 wt % Cr), (c) Ni_NP_@NiO_*x*_/BaLa_4_Ti_4_O_15_ (0.50 wt % Ni),^67^ and (d) Rh_2−*x*_Cr_*x*_O_3_(3.0 nm)/BaLa_4_Ti_4_O_15_ (0.10 wt % Rh and 0.15 wt % Cr) (Table 1).

The gas‐evolution rate of Rh_2−*x*_Cr_*x*_O_3_(1.3 nm)/BaLa_4_Ti_4_O_15_ containing 0.09 wt % Rh and 0.10 wt % Cr (Table 1) is shown in Figure 9 a; this photocatalyst exhibited the highest activity of the investigated photocatalysts (Figure S18). The ratio of the generated H_2_ and O_2_ amount was close to 2:1, indicating that the water‐splitting reaction proceeded ideally. The water‐splitting activity of this sample was approximately four times higher than that of BaLa_4_Ti_4_O_15_ loaded with Au_25_ clusters^57^, ^58^, ^59^, ^60^, ^61^, ^62^, ^63^, ^64^, ^65^, ^66^, ^67^ protected by a Cr_2_O_3_ film (Au_25_@Cr_2_O_3_/BaLa_4_Ti_4_O_15_)^42^, ^43^ (Figure 9 b and Table 1). Moreover, the Rh_2−*x*_Cr_*x*_O_3_(1.3 nm)/BaLa_4_Ti_4_O_15_ photocatalyst also showed higher water‐splitting activity than that of BaLa_4_Ti_4_O_15_ loaded with 0.50 wt % Ni (Ni_NP_@NiO_*x*_/BaLa_4_Ti_4_O_15_, Figure S22), which exhibited the highest water‐splitting activity of previously reported BaLa_4_Ti_4_O_15_ catalysts (Figure 9 c and Table 1).^49^ These results demonstrate that the use of Rh_2−*x*_Cr_*x*_O_3_ particles as a cocatalyst is also very effective for improving the water‐splitting activity of BaLa_4_Ti_4_O_15_.^45^, ^46^, ^47^, ^48^, ^68^, ^69^ Rh_2−*x*_Cr_*x*_O_3_(1.3 nm)/BaLa_4_Ti_4_O_15_ containing 0.09 wt % Rh and 0.10 wt % Cr showed an apparent quantum yield of 16 % under 270 nm excitation.

**Table 1:** Gas‐evolution rates over various photocatalysts.

Photocatalyst	Ratio of cocatalysts elements	Loading of cocatalysts	H_2_ [mmol h^−1^]	O_2_ [mmol h^−1^]
Rh_2−*x*_Cr_*x*_O_3_(1.3 nm)/BaLa_4_Ti_4_O_15_	0.09 wt % Rh and 0.10 wt % Cr	this method^[a]^	9.9	5.1
Au_25_@Cr_2_O_3_/BaLa_4_Ti_4_O_15_	0.10 wt % Au and 0.50 wt % Cr	our previous method^[b]^	2.4	1.2
Ni_NP_@NiO_*x*_/BaLa_4_Ti_4_O_15_	0.50 wt % Ni	impregnation	1.2	0.6
Rh_2−*x*_Cr_*x*_O_3_(3.0 nm)/BaLa_4_Ti_4_O_15_	0.10 wt % Rh and 0.15 wt % Cr	impregnation	7.3	3.6

[a] Photodeposition of Cr_2_O_3_+adsorption of Rh–SG complexes+calcination of photocatalysts. [b] Photodeposition of Cr_2_O_3_+adsorption of SG‐protected Au_25_ cluster+calcination of photocatalysts.^43^

To examine the importance of the preparation method used in this study, we also attempted to load the Rh_2−*x*_Cr_*x*_O_3_ particles onto BaLa_4_Ti_4_O_15_ by impregnation (Figure S23) and photodeposition methods (Figure S24). We found that these techniques could not form Rh_2−*x*_Cr_*x*_O_3_ particles with a size comparable to that achieved by the method developed in this study. Therefore, our technique is important because it allows loading of ultrafine Rh_2−*x*_Cr_*x*_O_3_ particles. The impregnation method allowed loading of Rh_2−*x*_Cr_*x*_O_3_ particles onto BaLa_4_Ti_4_O_15_ to give a photocatalyst with 0.10 wt % Rh and 0.15 wt % Cr with a particle size of 3.0±2.3 nm (Rh_2−*x*_Cr_*x*_O_3_(3.0 nm)/BaLa_4_Ti_4_O_15_; Figure S23). This photocatalyst showed the highest water‐splitting activity among the Rh_2−*x*_Cr_*x*_O_3_/BaLa_4_Ti_4_O_15_ photocatalysts prepared by the impregnation and photodeposition methods (Figure S24). However, the gas‐evolution rate of Rh_2−*x*_Cr_*x*_O_3_(3.0 nm)/BaLa_4_Ti_4_O_15_ was ≈74 % of that of Rh_2−*x*_Cr_*x*_O_3_(1.3 nm)/BaLa_4_Ti_4_O_15_ prepared by our technique (Figure 9 d and Table 1). These two photocatalysts were prepared via different methods, therefore, there may be additional differences other than the size of the Rh_2−*x*_Cr_*x*_O_3_ particles, such as the position of cocatalysts on the surface, that contribute to the higher water‐splitting activity of Rh_2−*x*_Cr_*x*_O_3_(1.3 nm)/BaLa_4_Ti_4_O_15_ compared with that of Rh_2−*x*_Cr_*x*_O_3_(3.0 nm)/BaLa_4_Ti_4_O_15_ (Figure S25). Overall, this study demonstrates that loading ultrafine Rh_2−*x*_Cr_*x*_O_3_ particles by the developed technique is an effective approach to improve the water‐splitting activity of BaLa_4_Ti_4_O_15_.

## Conclusion

We loaded ultrafine Rh_2−*x*_Cr_*x*_O_3_ particles with a size of approximately 1.3 nm and a narrow size distribution onto the BaLa_4_Ti_4_O_15_ photocatalyst by establishing a new loading method for the Rh_2−*x*_Cr_*x*_O_3_ particles. The obtained photocatalyst exhibited a remarkably high quantum yield for water splitting compared to that achieved for BaLa_4_Ti_4_O_15_ in previous studies. Although this study used only BaLa_4_Ti_4_O_15_ as the photocatalyst,^42^, ^43^ the developed loading method can, in principal, be applied to other photocatalysts as well. Rh_2−*x*_Cr_*x*_O_3_ particles are a useful cocatalyst for many water‐splitting photocatalysts,^45^, ^46^, ^47^, ^48^, ^68^, ^69^ therefore, we expect that this loading method should also be suitable to improve the water‐splitting activity of other advanced photocatalysts.

## Conflict of interest

The authors declare no conflict of interest.

## Supporting information

As a service to our authors and readers, this journal provides supporting information supplied by the authors. Such materials are peer reviewed and may be re‐organized for online delivery, but are not copy‐edited or typeset. Technical support issues arising from supporting information (other than missing files) should be addressed to the authors.

Supplementary
