# Supplementary material for: Activation of Water‐Splitting Photocatalysts by Loading with Ultrafine Rh–Cr Mixed‐Oxide Cocatalyst Nanoparticles
Source: Angew Chem Int Ed Engl. 2020 Mar 6;59(18):7076–82. doi: 10.1002/anie.201916681 (PMC7318701; doi:10.1002/anie.201916681)
Supplement: Supplementary file 1 — Supplementary [file ANIE-59-7076-s001.pdf]

Angewandte Chemie
Eine Zeitschrift der Gesellschaft Deutscher Chemiker

Supporting Information

Activation of Water-Splitting Photocatalysts by Loading with Ultrafine Rh–Cr Mixed-Oxide Cocatalyst Nanoparticles

*Wataru Kurashige, Yutaro Mori, Shuhei Ozaki, Masanobu Kawachi, Sakiat Hossain, Tokuhisa Kawawaki, Cameron J. Shearer, Akihide Iwase, Gregory F. Metha, Seiji Yamazoe, Akihiko Kudo, and Yuichi Negishi**

anie_201916681_sm_miscellaneous_information.pdf

1. Experimental Section

1.1. Chemicals

All of the chemicals were commercially obtained and used without further purification. Rhodium(III) chloride ($RhCl_3$) and hydrogen tetrachloroaurate tetrahydrate ($HAuCl_4 \cdot 4H_2O$) was purchased from Tanaka Kikinzoku. Glutathione (GSH), titanium tetrabutoxide ($Ti(OC_4H_9)_4$), lanthanum nitrate hexahydrate ($La(NO_3)_3 \cdot 6H_2O$), barium carbonate ($BaCO_3$), chromium(VI) oxide (CrO_3), potassium chromate (K_2CrO_4), tetraoctylammonium bromide ($(C_8H_{17})_4NBr$), triphenylphosphine ($(C_6H_5)_3P$), rhodium(III) nitrate nonahydrate ($Rh(NO_3)_3 \cdot 9H_2O$), propylene glycol, bismuth standard solution (100 ppm), chromium standard solution (1000 ppm), and rhodium standard solution (1000 ppm) were obtained from FUJIFILM Wako Pure Chemical CO.. Citric acid, nickel(II) nitrate hexahydrate ($Ni(NO_3)_2 \cdot 6H_2O$), ethanol, and chloroform purchased from Kanto Chemical Co., Inc. Sodium tetrahydroborate ($NaBH_4$) was purchased from Tokyo Kasei Co., Ltd. Chromium(III) nitrate nonahydrate ($Cr(NO_3)_3 \cdot 9H_2O$) was purchased from Nacalai Tesque, Inc. Gold powder was purchased from Inuisho Precious Metals. Pure Milli-Q water (18.2 MΩ·cm) was generated using a Merck Millipore Direct 3 UV system.

1.2. Synthesis

Synthesis of the Rh−SG complex. First, $RhCl_3$ (0.1 mmol) was added to water (100 mL) containing GSH (30 mg, 0.1 mmol). After stirring for 15 min, water (10 mL) containing $NaBH_4$ (40 mg, 1.0 mmol) was added. The solution was stirred for 2 h. Impurities such as excess GSH and reducing agent were then removed from the sample by ultrafiltration (MW 5000 filter) to give the Rh−SG complex.

$BaLa_4Ti_4O_{15}$. $BaLa_4Ti_4O_{15}$ photocatalyst was prepared by a polymerizable complex method.[1,2] First, $Ti(OC_4H_9)_4$ (6.06 g, 17.8 mmol) and propylene glycol (60.9 g, 800 mmol) were added to ethanol (30 mL), and then the solution was heated to 70 °C. Then, citric acid (838 mg, 200 mmol), $La(NO_3)_3 \cdot 6H_2O$ (7.70 g, 17.8 mmol), and $BaCO_3$ (878 mg, 4.45 mmol) were sequentially added, and then the solution was heated at 120−130 °C for 6 h. After grinding the obtained gray powder, it was transferred to an electric furnace and heated at 500 °C. After cooling and grinding, its powder was transferred to the electric furnace and heated at 1100 °C. About 5.0 g of $BaLa_4Ti_4O_{15}$ was obtained by this preparation method.

$Rh_{0.5}Cr_{1.5}O_3$. $Rh_{0.5}Cr_{1.5}O_3$ was synthesized according to the reported method.[3] First, $Cr(NO_3)_3 \cdot 9H_2O$ (168 mg, 0.70 mmol) and $Rh(NO_3)_3 \cdot 9H_2O$ (56.0 mL, 0.14 mmol) were put in an alumina crucible, and it was well dispersed with ultrasonic waves. Thereafter it was calcined at 150 °C for 12 h, 400 °C, 24 h and 1000 °C, 48 h to give $Rh_{0.5}Cr_{1.5}O_3$.

$Au_{25}(SG)_{18}$. $Au_{25}(SG)_{18}$ was synthesized by the conversion of $(C_6H_5)_3P$-protected Au_n clusters into $Au_{25}(SG)_{18}$ through a ligand-exchange reaction.[4] $(C_6H_5)_3P$-protected Au_n clusters were prepared by a procedure similar to the method reported by Jin and co-workers.[5] Chloroform (75 mL) containing $(C_6H_5)_3P$-protected Au_n clusters (50.4 mg, 1.15 μmol) was mixed with water (75 mL) containing GSH (1.46 g, 4.70 mmol). The mixture was heated under reflux at 60 °C for about 10 h. The aqueous phase was then separated from the chloroform phase using a separation funnel. High-purity $Au_{25}(SG)_{18}$ was obtained by removing excess GSH by ultrafiltration of the aqueous phase.

1.3. Preparation of photocatalysts

$Rh_{2-x}Cr_xO_3/BaLa_4Ti_4O_{15}$. A Cr_2O_3 layer was loaded on $BaLa_4Ti_4O_{15}$ (Figure 1a) by photodeposition (Figure

1(b)).[6] In this process, $BaLa_4Ti_4O_{15}$ photocatalyst (650 mg) was added to an aqueous K_2CrO_4 solution (350 mL) in a quartz cell. The mixing ratio of K_2CrO_4 to $BaLa_4Ti_4O_{15}$ was changed within the range of 0.05−0.20 wt% Cr (Figure S18). After removing dissolved air by Ar bubbling, the suspension was irradiated with a high-pressure Hg lamp (400 W) under an Ar flow of 30 mL/min at 25 °C for 1.5 h. The actual amounts of Cr loaded on $BaLa_4Ti_4O_{15}$ were determined by inductively coupled plasma mass spectrometry (ICP-MS) of the aqueous solution after mixing. The solid $Cr_2O_3/BaLa_4Ti_4O_{15}$ was collected by centrifugation, washed with water three times, and dried by evaporation.

The Rh−SG complex was adsorbed on $Cr_2O_3/BaLa_4Ti_4O_{15}$ by mixing an aqueous solution of the Rh−SG complex with an aqueous solution of $BaLa_4Ti_4O_{15}$ (600 mg) for 2 h at room temperature (Figure 1c). The total volume of aqueous solution was fixed at 200 mL. The actual amount of Rh adsorbed on $BaLa_4Ti_4O_{15}$ was determined by ICP-MS of the aqueous solution after mixing (Table S2).

The obtained Rh−SG/$Cr_2O_3/BaLa_4Ti_4O_{15}$ (550 mg) was calcined under reduced pressure (>1.0×10^{-1} Pa). The furnace temperature was increased at a rate of 7 °C/min and then maintained at 300 °C for 80 min to give $Rh_{2-x}Cr_xO_y/BaLa_4Ti_4O_{15}$ (Figure 1d).

$Rh_{2-x}Cr_xO_3$(1.3 nm)/$BaLa_4Ti_4O_{15}$ was obtained by irradiating $Rh_{2-x}Cr_xO_y/BaLa_4Ti_4O_{15}$ with a high-pressure Hg lamp (400 W) in aqueous solution for 1 h (Figure 1e).

Au_{25}@$Cr_2O_3/BaLa_4Ti_4O_{15}$. First, $Au_{25}(SG)_{18}$ was dissolved in water and $Cr_2O_3/BaLa_4Ti_4O_{15}$ (600 mg) was added to this solution (the total solution volume was fixed at 200 mL). This solution was stirred for 2 h, and then $Au_{25}(SG)_{18}/Cr_2O_3/BaLa_4Ti_4O_{15}$ was obtained by centrifugation and evaporation. The actual amount of Au adsorbed on $Cr_2O_3/BaLa_4Ti_4O_{15}$ was determined by ICP-MS analysis of the aqueous solution after mixing. The obtained $Au_{25}(SG)_{18}/Cr_2O_3/BaLa_4Ti_4O_{15}$ (550 mg) was calcined under reduced pressure (>1.0×10^{-1} Pa) at 300 °C for 80 min (7 °C/min) to give Au_{25}@Cr_2O_3*/$BaLa_4Ti_4O_{15}$. Au_{25}@$Cr_2O_3/BaLa_4Ti_4O_{15}$ was obtained by irradiating Au_{25}@Cr_2O_3*/$BaLa_4Ti_4O_{15}$ with a high-pressure Hg lamp (400 W) in aqueous solution for 1 h.[6]

Ni_{NP}@$NiO_x/BaLa_4Ti_4O_{15}$. Ni_{NP}@$NiO_x/BaLa_4Ti_4O_{15}$ was prepared by impregnation method. First, $BaLa_4Ti_4O_{15}$ impregnated with $Ni(NO_3)_2\cdot6H_2O$ was calcined at 270 °C for 1 h in air. Then, pretreatment of reduction with 200 Torr of H_2 at 400 °C for 2 h followed by oxidation with 100 Torr of O_2 at 100 °C for 1 h was carried out for NiO_x-loaded photocatalysts.[2]

$Rh_{2-x}Cr_xO_3/BaLa_4Ti_4O_{15}$ (impregnation method). $Rh_{2-x}Cr_xO_3/BaLa_4Ti_4O_{15}$ was also prepared by impregnation method.[7] First, $BaLa_4Ti_4O_{15}$ (600 mg) and water (4−5 mL) containing an appropriate amount of $RhCl_3$ and $Cr(NO_3)_3\cdot9H_2O$ were transferred into an evaporation dish that was subsequently placed in a steam bath. The suspension was stirred using a glass rod until the water was completely evaporated, and the resulting powder was collected and heated in air. The furnace temperature was increased at a rate of 7 °C/min and then maintained at 350 °C for 1 h.

$Rh_2O_3/BaLa_4Ti_4O_{15}$. $Rh_2O_3/BaLa_4Ti_4O_{15}$ was prepared by impregnation method.[7] First, $BaLa_4Ti_4O_{15}$ (600 mg) and water (4−5 mL) containing an appropriate amount of $RhCl_3$ were transferred into an evaporation dish that was subsequently placed in a steam bath. The suspension was stirred using a glass rod until the water was completely evaporated, and the resulting powder was collected and heated in air. The furnace temperature was increased at a rate of 7 °C/min and then maintained at 350 °C for 1 h.

$Rh_{NP}/BaLa_4Ti_4O_{15}$. Rh_{NP} was loaded on $BaLa_4Ti_4O_{15}$ by photodeposition method.[8] First, $BaLa_4Ti_4O_{15}$ photocatalyst (600 mg) was added to an aqueous $RhCl_3$ solution (350 mL) in a quartz cell. After removing dissolved air by evacuation through Ar bubbling, the suspension was irradiated with a high-pressure Hg lamp

(400 W) under Ar flow of 30 mL/min at 25 °C for 1.5 h. The solid $Rh_{NP}/BaLa_4Ti_4O_{15}$ was collected by centrifugation and then washed with water 3 times and subsequently dried by evaporation.

***Rh_{NP}@$Cr_2O_3/BaLa_4Ti_4O_{15}$*.** Cr_2O_3 was loaded on $Rh_{NP}/BaLa_4Ti_4O_{15}$ by photodeposition method.[8] First, $Rh_{NP}/BaLa_4Ti_4O_{15}$ photocatalyst (550 mg) was added to an aqueous K_2CrO_4 solution (350 mL) in a quartz cell. After removing dissolved air by evacuation through Ar bubbling, the suspension was irradiated with a high-pressure Hg lamp (400 W) under Ar flow of 30 mL/min at 25 °C for 1.5 h. The solid Rh_{NP}@$Cr_2O_3/BaLa_4Ti_4O_{15}$ was collected by centrifugation and then washed with water 3 times and subsequently dried by evaporation.

1.4. Characterization

The ultraviolet-visible absorption spectra were collected by a spectrometer (JASCO, V-670 or Shimadzu, UV 3600). The wavelength-dependent optical data ($I(w)$) were converted to energy-dependent data ($I(E)$) by the following equation, which conserved the integrated spectral areas: $I(E) = I(w)/|\partial E/\partial w| \propto I(w) \times w^2$.

The Fourier transform infrared spectrometer (FT-IR) attenuated total reflection (ATR) spectra were recorded in the region between 500 and 5000 cm^{-1} using a spectrometer (JASCO FT/IR-4600-ATR-PRO ONE) equipped with a DLATGS detector as the average of 50 scans at 4 cm^{-1} resolution.

Rh K- and Cr K-edge X-ray absorption fine structure (XAFS) measurements were performed at beamlines BL01B1 and BL37XU at the SPring-8 facility of the Japan Synchrotron Radiation Research Institute (proposal number 2018A0910, 2018A0919, and 2018B1422). The incident X-ray beam was monochromatized by Si (111), for Cr K edge, and Si(311), for Rh K edge, double-crystal monochromator, respectively. XAFS spectra of $Rh_{0.5}Cr_{1.5}O_3$, CrO_3, Cr_2O_3 and Cr foil (Cr K edge), and $Rh_{0.5}Cr_{1.5}O_3$, Rh_2O_3 and Rh foil (Cr K edge) as references were recorded in transmission mode using ionization chambers. Rh K-edge and Cr K-edge XAFS spectra for photocatalyst samples were measured in fluorescence mode using a 19-element Ge solid-state detector at room temperature. The X-ray energy was calibrated for the Rh K- and Cr K-edges using Rh foil and Cr_2O_3, respectively. The X-ray absorption near-edge structure (XANES) and extended X-ray absorption fine structure (EXAFS) spectra were analyzed using the xTunes program (https://doi.org/10.1016/j.radphyschem.2019.04.020) as follows. The χ spectra were extracted by subtracting the atomic absorption background by cubic spline interpolation and normalized to the edge height. The normalized data were used as XANES spectra. The k^3-weighted χ spectra within the k range of 3.0-13.0 $Å^{-1}$ for the Rh K-edge were Fourier transformed into r-space for structural analysis. The curve fitting analysis was carried out for Rh−O bonds over the r range of 1.2−1.9 Å in the Rh K-edge Fourier transform (FT)-EXAFS spectra. In the curve fitting analysis, the phase shifts and backscattering amplitude functions of Rh−O were extracted from Rh_2O_3 using the FEFF8 program.[9]

Electrospray ionization (ESI) mass spectrometry was performed using a reflectron time of flight mass spectrometer (Bruker, micrOTOF II). In the measurements, a Rh−SG solution with a concentration of 10−200 μg/mL in a mixture of water and methanol was electrosprayed at a flow rate of 180 μL/h.

Dynamic light scattering analyses were conducted using a spectrometer (Malvern Zetasizer-Nano ZSP) at 633 nm and a scattering angle of 173°. Recorded data were analyzed by BIC software (Brookhaven Instruments, Holtsville, NY, USA) and results were obtained from the mean radius of the log normal size distribution.

The X-ray photoelectron spectroscopy (XPS) data were collected by using an electron spectrometer (JEOL, JPS-9010MC) at a base pressure of ~2×10^{-8} Torr. X-rays from the Mg-Kα line (1253.6 eV) were used for excitation. The spectra were calibrated with the peak energies of Au 4f 7/2 (83.7 eV).

ICP-MS was performed with an Agilent 7500c spectrometer (Agilent Technologies, Tokyo, Japan). Bi was used as the internal standard.

The high-angle annular dark field scanning transmission electron microscope (HAADF-STEM) images were obtained by ultra-high-resolution transmission electron microscope (The FEI Titan Themis 80−200) operating at 200 kV, with a beam convergence semi angle of 25 mrad and HAADF collection angle from 56−200 mrad. Elemental maps were acquired using a super X detector and low-background sample holder.

The transmission electron microscope (TEM) images were recorded with a JEM-2100 electron microscope (JEOL) operating at 200 kV, typically using magnification of 400,000−600,000.

The X-ray diffraction (XRD) patterns of the samples were measured with a Rint2500 diffractometer (Rigaku) using Cu-Kα source operated at 50 kV and 100 mA. A reflection-free silicon plate was used as a substrate.

Scanning electron microscope (SEM) images ware recorded with a JSM-7600F (JEOL), typically using magnification of 10000.

1.5. Measurement of photocatalytic activity

Water-splitting. The photocatalytic water-splitting reaction was performed at room temperature using an experimental apparatus built in-house consisting of a high-pressure Hg lamp (400 W) and quartz cell.[6] The reaction was performed with an Ar gas flow rate of 30 mL/min. Before the measurements, the reaction solution containing the prepared photocatalyst (500 mg) in mili-Q water (350 mL) was purged with Ar gas for 1 h to ensure complete removal of air from the reaction vessel. The apparent quantum yield at 270 nm was estimated using a top-irradiation cell made of quartz and 300-W Xe lamp (Asahi Spectra; MAX-302) equipped with band-pass filter. The number of incident photons was determined using a photodiode (Ophir: PD300-UV head and NOVA power monitor). The photocatalyst powder (100 mg) was dispersed in pure water (300 mL) to measure the apparent quantum yield.

O_2-photoreduction reaction. The decrease of the amount of evolved H_2 was examined to investigate the likelihood of the O_2-photoreduction reaction. In particular, the photocatalytic water-splitting reaction was investigated under a gas flow consisting of a 7:3 mixture of Ar to air instead of pure Ar. Under these experimental conditions, sufficient O_2 is included in the reaction system for the O_2-photoreduction reaction to occur.

Back reaction. The back reaction between H_2 and O_2 to produce H_2O in the gas phase in the dark was examined using a gas-tight circulation system with a dead volume of 400–500 mL. In this experiment, 100 mg of photocatalyst was used.

1.6. Estimation of the constitute metal atoms included in $Rh_{2-x}Cr_xO_3$ particles

Assuming that $Rh_{2-x}Cr_xO_3$ particles are supported hemispherically on the photocatalyst as M_2O_3 (M = Rh or Cr), the number of total metal atoms in the particle can be obtained from equations 1 and 2.

$$n = \frac{V \times \rho \times N_A}{M_w} \times 2 \quad (1)$$

$$V = \frac{4\pi}{3}\left(\frac{D}{2}\right)^3 \times \frac{1}{2} \quad (2)$$

Here, n, ρ, N_A, M_W, and D indicate the number of total metal atoms, density of M_2O_3, Avogadro constant, molecular weight of M_2O_3, and particle diameter, respectively. The density of M_2O_3 and the molecular weight of M_2O_3 were changed in the range of Rh:Cr = 1:4−4:1 and thereby the number of Rh atoms in each $Rh_{2-x}Cr_xO_3$ particle was estimated (Figure S15).

2. Additional Tables

Table S1. Assignments of ESI Mass Spectrum

m/z	peak [a]	Assignment
611.1		$[GSSG-H]^-$
633.1		$[GSSG-2H+Na]^-$
685.9		$[Rh^{I}Rh^{II}(SG)_2-COC_3H_5(NH_2)COOH-2H]^-$
707.8		$[Rh^{I}Rh^{II}(SG)_2-2COOH-3H-OH]^-$
768.9		$[Rh^{II}Rh^{III}(SG)_2-4H-COOH]^-$
812.9	ii	$[Rh^{III}_2(SG)_2-5H]^-$
814.9	i	$[Rh^{II}_2(SG)_2-3H]^-$
836.9		$[Rh^{II}_2(SG)_2-4H+Na]^-$
948.8		$[Rh^{I}_2(SG)_2-2H+Cs]^-$

[a] see Figure 3(d).

Table S2. Adsorption Rate and Actual Amount of Rh on Cr_2O_3/$BaLa_4Ti_4O_{15}$ Including 0.10 wt% Cr

Rh (wt%)	Adsorption rate (%) [a]	Loading amount (wt%)
0.05	99	0.05
0.10	89	0.09
0.15	87	0.13

[a] These values were estimated by ICP-MS analysis of each aqueous solution after mixing.

Table S3. Curve Fitting Analysis for Each Sample

Sample	Bond	C.N. [a]	R (Å)	D.W. [b]	R factor (%)
$Rh_{2-x}Cr_xO_y$/$BaLa_4Ti_4O_{15}$ (after calcination)	Rh–O	4.0 (2)	2.040 (3)	0.0018 (2)	5.0
$Rh_{2-x}Cr_xO_3$/$BaLa_4Ti_4O_{15}$ (after irradiation)	Rh–O	3.9 (2)	2.028 (3)	0.0019 (2)	7.6
$Rh_{1.5}Cr_{0.5}O_3$	Rh–O	5.1 (2)	2.036 (3)	0.0024 (2)	5.5
Rh_2O_3	Rh–O	4.8 (2)	2.057 (3)	0.0024 (2)	7.8

The numbers in parentheses are uncertainties; 4.0(2) and 2.040(3) represent 4.0 ± 0.2 and 2.040 ± 0.003, respectively.
[a] Coordination number. [b] DW = Debye–Waller factor.

3. Additional Scheme

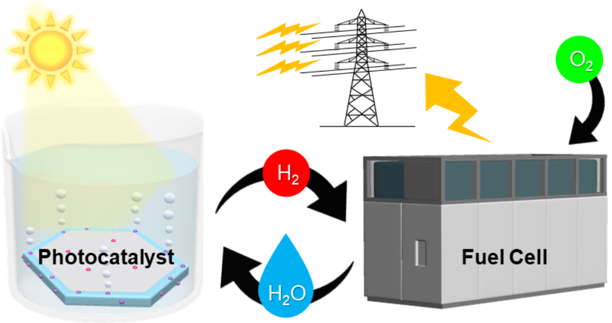

Scheme S1. Potential future energy conversion system consisting of photocatalysts and fuel cell to produce electrical energy without consuming fossil fuels and producing carbon dioxide.[10]

4. Additional Figures

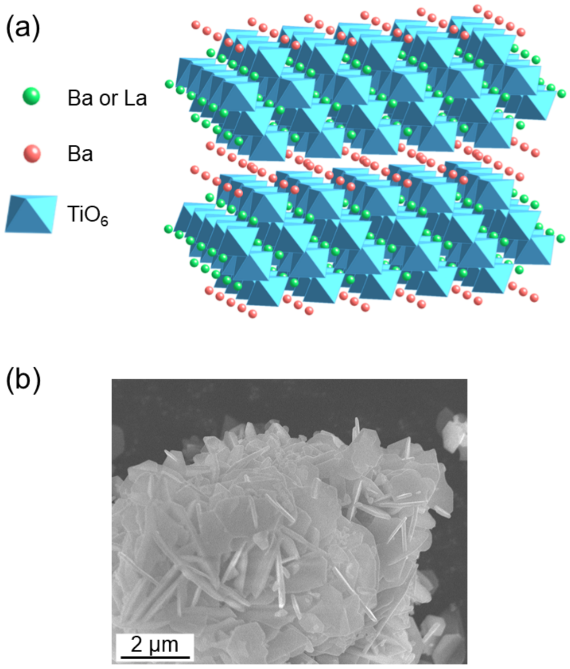

Figure S1. (a) Geometrical structure of $BaLa_4Ti_4O_{15}$. (b) SEM image of the prepared $BaLa_4Ti_4O_{15}$.

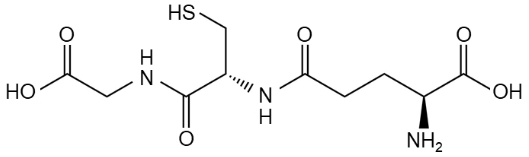

Figure S2. Geometrical structure of GSH. SG is the form in which H of SH is deprotonated.

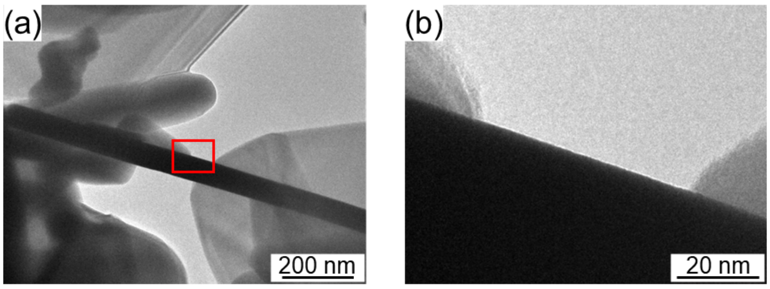

Figure S3. High resolution-TEM images of Cr_2O_3/$BaLa_4Ti_4O_{15}$ observed at (a) low and (b) high magnification for a flat surface. In this experiment, Cr was loaded at 1 wt% for easily monitoring the position of Cr_2O_3 layers. The Cr_2O_3 layers were negligibly observed at the surface of $BaLa_4Ti_4O_{15}$, indicating that the Cr_2O_3 layers are formed preferentially at the edge of $BaLa_4Ti_4O_{15}$ (Figure 2).

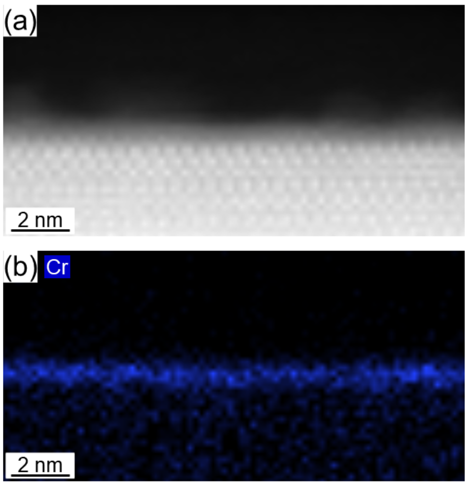

Figure S4. (a) STEM image and (b) elemental mapping of Cr obtained for Rh–SG/Cr_2O_3/$BaLa_4Ti_4O_{15}$ (Figure 1(c)). The surface without the adsorption of Rh–SG complex is selected in this figure, in contrast to Figure 4B(a) and 4C(a). As can be seen, the Cr_2O_3 layer is mainly loaded at the edge of $BaLa_4Ti_4O_{15}$ by the photodeposition method, because the reduction of the metal ions occurs easier at the edge of $BaLa_4Ti_4O_{15}$ than at the flat surface of $BaLa_4Ti_4O_{15}$.[2] This means that the $Rh_{2-x}Cr_xO_3$ particles could also be formed preferentially at the edge of $BaLa_4Ti_4O_{15}$ (Figure S25(a)), which is the different from the case of the loading of $Rh_{2-x}Cr_xO_3$ particles by impregnation methods (Figure S25(b)).[7]

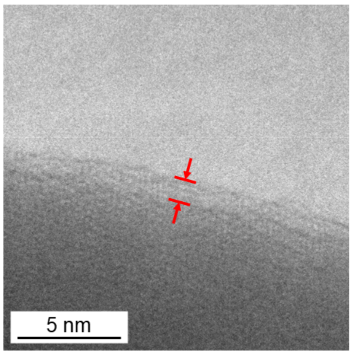

Figure S5. HR-TEM image of $Cr_2O_3/BaLa_4Ti_4O_{15}$ with 0.1 wt% Cr observed at high magnification for the edge of $BaLa_4Ti_4O_{15}$.

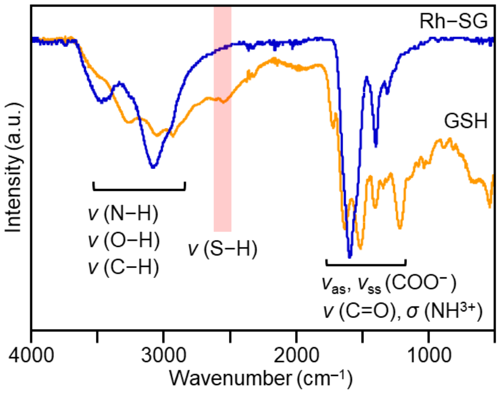

Figure S6. FT-IR spectrum of Rh−SG complex together with that of GSH. In figure, "ν", "ν_{as}", "ν_{ss}", and "σ" indicate the stretching vibration, asymmetric stretching vibration, symmetric stretching vibration, and in plane bending vibration, respectively.[11−13]

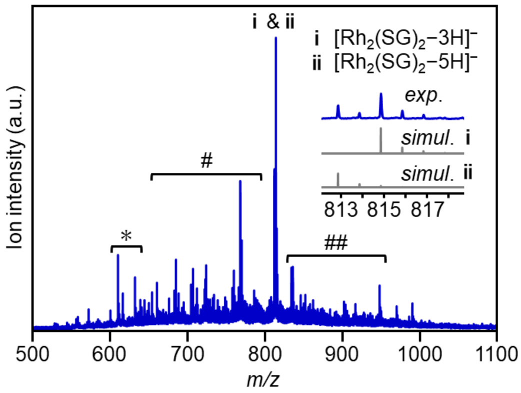

Figure S7. Assignments of ESI mass spectrum of Rh−SG complex. As shown in Figure 3(d), the main peak group (**i** and **ii**) is composed of $Rh_2(SG)_2$ containing Rh(II) (**i**) or Rh(III) (**ii**). The sharp peaks (# and ##) are due to the fragments and adducts of $Rh_2(SG)_2$, respectively. The asterisk peaks (*) are due to the species which do not include Rh, $(GSH)_2$. The assignments of the peaks *, #, and ## are summarized in Table S1.

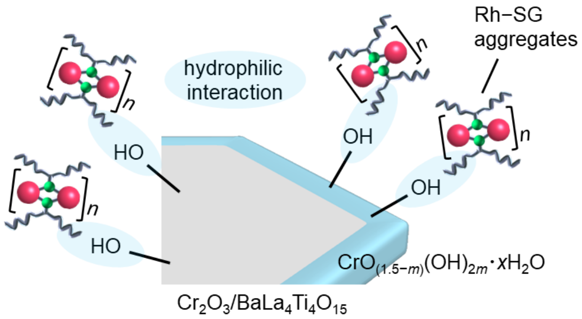

Figure S8. Proposed sites and interaction for the adsorption of Rh−SG complex on $Cr_2O_3/BaLa_4Ti_4O_{15}$. Since the surfaces of both Cr_2O_3 and bare $BaLa_4Ti_4O_{15}$ include −OH groups, Rh−SG complexes should be adsorbed on both Cr_2O_3 and bare surface of $BaLa_4Ti_4O_{15}$. On the basis of the diameter of $Rh_{2-x}Cr_xO_3$ particles observed in the TEM image of $Rh_{2-x}Cr_xO_3$(1.3 nm)/$BaLa_4Ti_4O_{15}$ (Figure 6C(b)), the Rh−SG complex is estimated to be conglomerated on the surface (Figure 4B(a) and 4C(a)(c)), forming Rh−SG aggregates, $[Rh_2(SG)_2]_n$ (Figure S15).

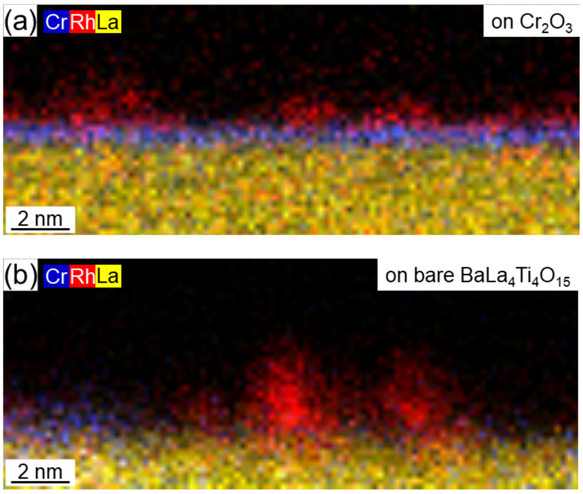

Figure S9. STEM-EDX elemental mappings of Rh−SG/$Cr_2O_3/BaLa_4Ti_4O_{15}$. In (a), Rh−SG complexes are adsorbed on Cr_2O_3 (Figure 1(c)), whereas in (b) Rh−SG complexes are adsorbed on bare surface of BaLa-$_4Ti_4O_{15}$. This result indicates that Rh−SG complexes can be adsorbed on both Cr_2O_3 and bare surface of $BaLa_4Ti_4O_{15}$ (Figure S8). This result also support the interpretation that the Rh−SG complexes are conglomerated on the surface (Figure 4B(a) and 4C(a)(c)), forming Rh−SG aggregates, $[Rh_2(SG)_2]_n$ (Figure S8).

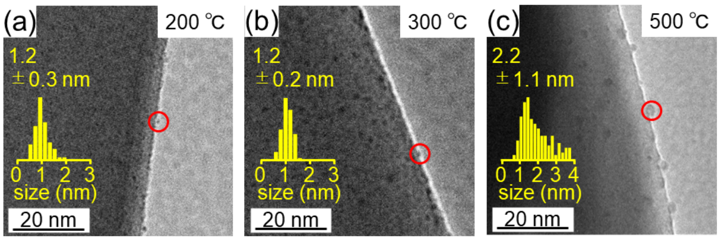

Figure S10. Relation between calcination temperature and the sizes of $Rh_{2-x}Cr_xO_3$ particles obtained for $Rh_{2-x}Cr_xO_3/BaLa_4Ti_4O_{15}$ photocatalysts including 0.09 wt% Rh and 0.10 wt% Cr; (a) 200, (b) 300, and (c) 500 °C. These results indicate that the apparent aggregation of the particles occurred at the temperature of 500 °C during the calcination.

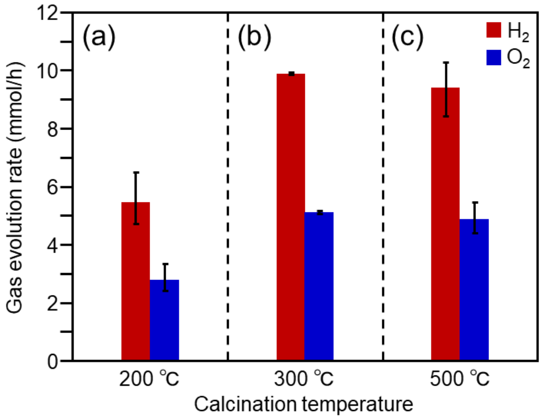

Figure S11. Relation between calcination temperature and the water-splitting activity obtained for $Rh_{2-x}Cr_xO_3/BaLa_4Ti_4O_{15}$ photocatalysts including 0.09 wt% Rh and 0.10 wt% Cr (Figure S10). These results indicate that the apparent aggregation of the particles (Figure S10(c)) leads to the decrease of water-splitting activity ((c)). The relation between the particles size and the water-splitting activity is consistent with that obtained from the comparison between $Rh_{2-x}Cr_xO_3$(1.3 nm)/$BaLa_4Ti_4O_{15}$ and $Rh_{2-x}Cr_xO_3$(3.0 nm)/$BaLa_4Ti_4O_{15}$ (Figure 9(a) vs. 9(d)).

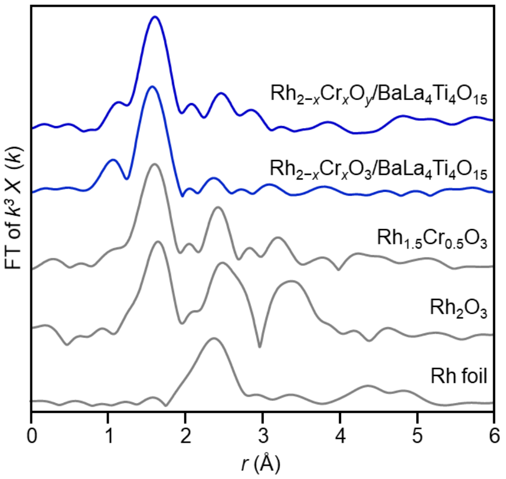

Figure S12. Comparison of Rh K-edge FT-EXAFS spectra between $Rh_{2-x}Cr_xO_y/BaLa_4Ti_4O_{15}$ (Figure 1(d)) and $Rh_{2-x}Cr_xO_3/BaLa_4Ti_4O_{15}$ (Figure 1(e)) together with those of Rh foil, Rh_2O_3, and $Rh_{0.5}Cr_{1.5}O_3$. Coordination numbers of Rh−O bonds estimated for $Rh_{2-x}Cr_xO_y/BaLa_4Ti_4O_{15}$, $Rh_{2-x}Cr_xO_3/BaLa_4Ti_4O_{15}$, Rh_2O_3, and $Rh_{0.5}Cr_{1.5}O_3$ are listed in Table S3.

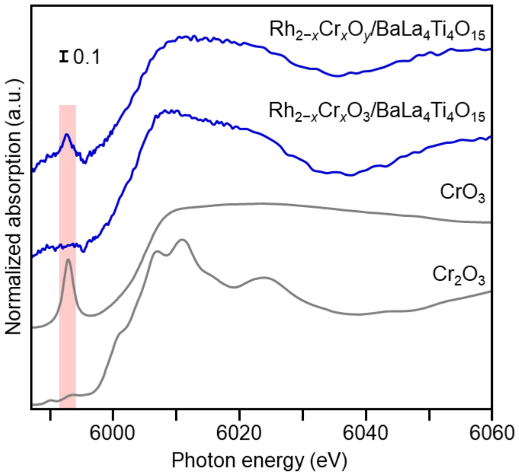

Figure S13. Comparison of Cr K-edge XANES spectra between $Rh_{2-x}Cr_xO_y/BaLa_4Ti_4O_{15}$ (Figure 1(d)), $Rh_{2-x}Cr_xO_3/BaLa_4Ti_4O_{15}$ (Figure 1(e)), CrO_3, and Cr_2O_3. In the Cr K-edge XANES spectra of $Rh_{2-x}Cr_xO_y/BaLa_4Ti_4O_{15}$, a weak peak exists at ~5992 eV similar to that of CrO_3. This peak is attributed to tetrahedral chromium oxide species, which is characteristic of chromium oxides with a high degree of oxidation, indicating that some of the chromium oxide was further oxidized during calcination. This peak disappeared after the light irradiation, indicating that chromium oxides with a high degree of oxidation was reduced by the light irradiation to Cr(III).

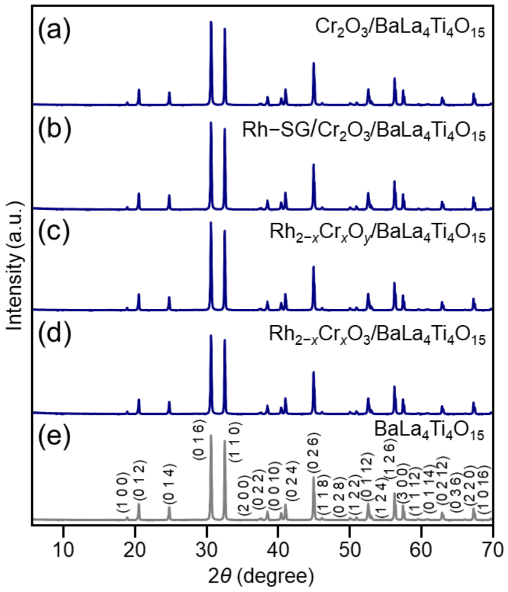

Figure S14. Comparison of XRD patterns for (a) $Cr_2O_3/BaLa_4Ti_4O_{15}$ (Figure 1(b)), (b) Rh–SG/$Cr_2O_3/BaLa_4Ti_4O_{15}$ (Figure 1(c)), (c) $Rh_{2-x}Cr_xO_y/BaLa_4Ti_4O_{15}$ (Figure 1(d)), and (d) $Rh_{2-x}Cr_xO_3/BaLa_4Ti_4O_{15}$ (Figure 1(e)) together with that of (e) $BaLa_4Ti_4O_{15}$ for comparison. The PDF number of $BaLa_4Ti_4O_{15}$ is 01–073–7800.

diameter of $Rh_{2-x}Cr_xO_3$ particle	total metal atoms			Rh atoms		
	Cr:Rh= 1:4	1:1	4:1	1:4	1:1	4:1
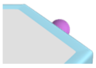 1.0 nm	10.3	10.4	10.6	8.2	5.2	2.1
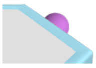 1.3 nm	22.6	23.0	23.4	18.1	11.5	4.7
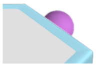 1.6 nm	42.1	42.8	43.6	33.7	21.4	8.7

Figure S15. Tentative counting of the number of constitute Rh atoms in one $Rh_{2-x}Cr_xO_3$ particle of $Rh_{2-x}Cr_xO_3/BaLa_4Ti_4O_{15}$ (Figure 1(e)). It is estimated that $Rh_{2-x}Cr_xO_3$ particle of 1.3 nm includes around 24 metal atoms on the basis of the densities of bulk Rh_2O_3 and Cr_2O_3 and the atomic weights of Rh, Cr, and O (see section 1.6 in the supporting information). If each $Rh_{2-x}Cr_xO_3$ particle would contain the same number of Rh and Cr, the number of Rh atoms in each $Rh_{2-x}Cr_xO_3$ particle of 1.3 nm is estimated to be around twelve. This means around six $Rh_2(SG)_2$ gathered in one Rh−SG aggregate of Rh–SG/$Cr_2O_3/BaLa_4Ti_4O_{15}$ (Figure 1(c)). However, at present, the exact chemical composition of $Rh_{2-x}Cr_xO_3$ particle was not elucidated.

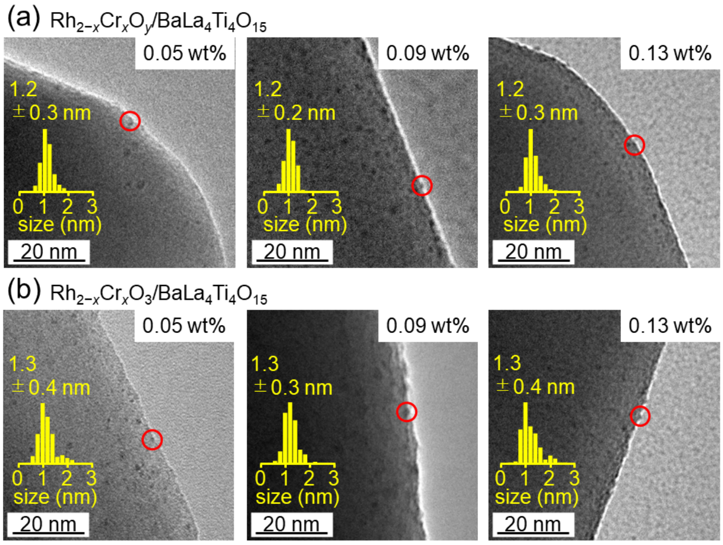

Figure S16. TEM images for (a) $Rh_{2-x}Cr_xO_y/BaLa_4Ti_4O_{15}$ (Figure 1(d)) and (b) $Rh_{2-x}Cr_xO_3/BaLa_4Ti_4O_{15}$ (Figure 1(e)). In these experiments, the loading weight of Cr was 0.10 wt%. The red circles indicate the $Rh_{2-x}Cr_xO_y$ or $Rh_{2-x}Cr_xO_3$ particles. These results indicate that the average particle sizes do not depend on the loading weight of Rh in the range of 0.05−0.13 wt%.

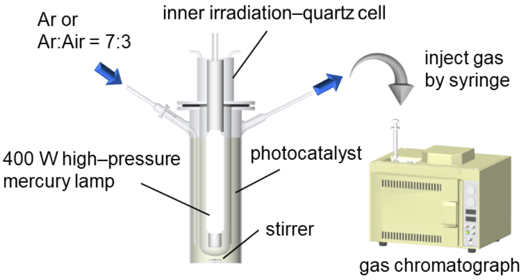

Figure S17. Schematic of the system used to estimate photocatalytic activity in this study.

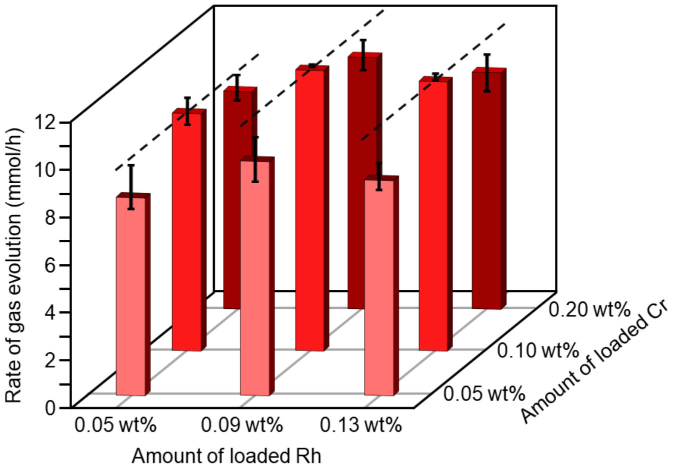

Figure S18. Relation between the loading amounts (wt%) of Rh and Cr and the gas evolution rate obtained for $Rh_{2-x}Cr_xO_3$(1.3 nm)/$BaLa_4Ti_4O_{15}$ prepared by this work's method (Figure S16). $Rh_{2-x}Cr_xO_3$(1.3 nm)/$BaLa_4Ti_4O_{15}$ including 0.09 wt% Rh and 0.10 wt% Cr exhibited the highest activity.

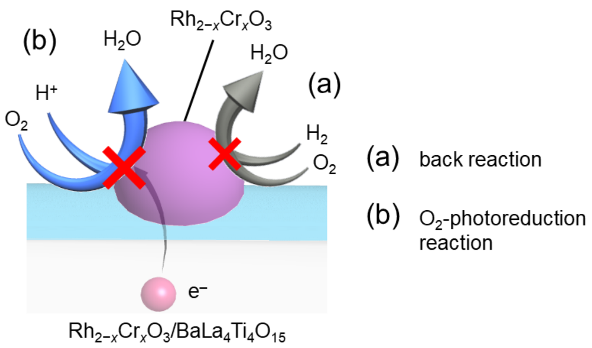

Figure S19. Possible reverse reactions that occur on $Rh_{2-x}Cr_xO_3$ particles of $Rh_{2-x}Cr_xO_3$/$BaLa_4Ti_4O_{15}$ during photocatalytic reaction: (a) back reaction and (b) O_2-photoreduction reaction. Both reactions were well suppressed on $Rh_{2-x}Cr_xO_3$ particles, as shown in Figure S20 and S21.

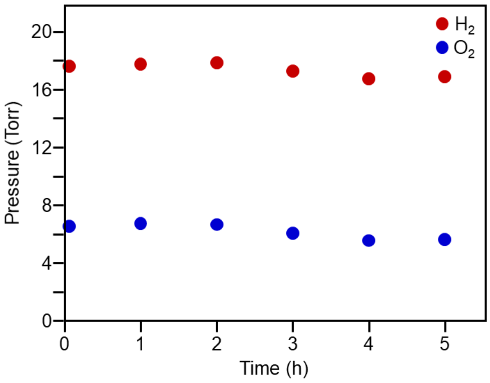

Figure S20. Measurement of the back reaction between H_2 and O_2 to produce H_2O (Figure S19(a)) in the gas phase in the dark over $Rh_{2-x}Cr_xO_3$(1.3 nm)/$BaLa_4Ti_4O_{15}$ including 0.09 wt% Rh and 0.10 wt% Cr. This experiment was conducted in the gas phase in the dark using a gas-tight circulation system with a dead volume of 400–500 mL. Both H_2 and O_2 were hardly reduced for 5 h, indicating that the back reaction (Figure S19(a)) is relatively well suppressed on $Rh_{2-x}Cr_xO_3$(1.3 nm)/$BaLa_4Ti_4O_{15}$ including 0.09 wt% Rh and 0.10 wt% Cr.

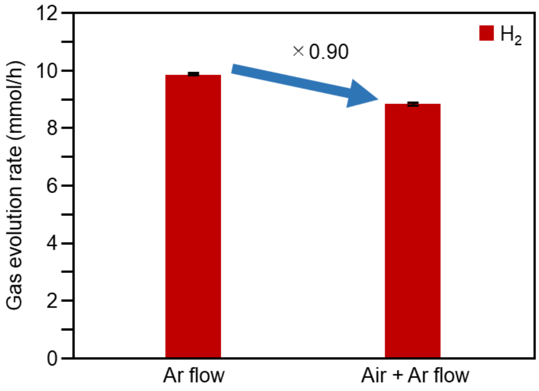

Figure S21. Comparison of the rates of photocatalytic generation of H_2 (a) under Ar flow and (b) under a flow of 7:3 mixture of Ar to air over $Rh_{2-x}Cr_xO_3$ (1.3 nm)/$BaLa_4Ti_4O_{15}$ including 0.09 wt% Rh and 0.10 wt% Cr. Averages of the values obtained from several experiments are used in this figure. This result indicates that O_2-photoreduction reaction (Figure S19(b)) is relatively well suppressed on $Rh_{2-x}Cr_xO_3$(1.3 nm)/$BaLa_4Ti_4O_{15}$ including 0.09 wt% Rh and 0.1 wt% Cr.

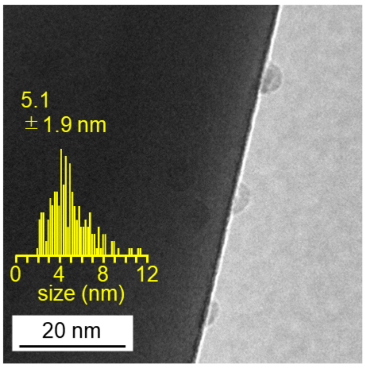

Figure S22. TEM image of Ni_{NP}@NiO_x/$BaLa_4Ti_4O_{15}$ including 0.50 wt% Ni. This sample was prepared by impregnation method.[2]

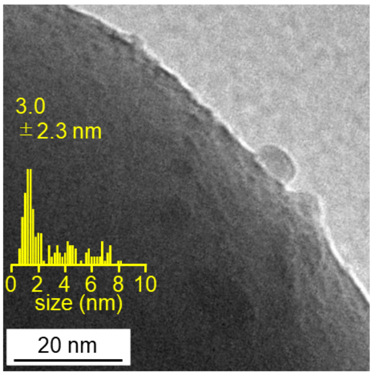

Figure S23. TEM image of $Rh_{2-x}Cr_xO_3$(3.0 nm)/$BaLa_4Ti_4O_{15}$ including 0.10 wt% Rh and 0.15 wt% Cr prepared by impregnation method.

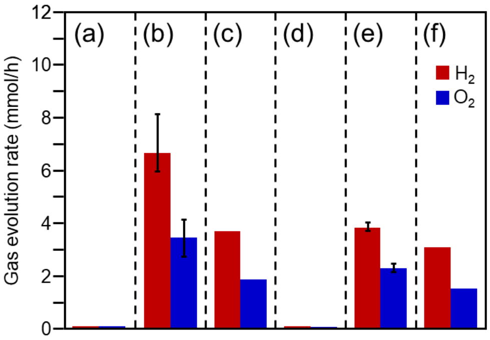

Figure S24. Comparison of water-splitting activity between (a) $Rh_2O_3/BaLa_4Ti_4O_{15}$ (0.10 wt% Rh), (b) $Rh_{2-x}Cr_xO_3/BaLa_4Ti_4O_{15}$ (0.10 wt% Rh and 0.15 wt% Cr), (c) $Rh_{2-x}Cr_xO_3/BaLa_4Ti_4O_{15}$ (1.0 wt% Rh and 1.5 wt% Cr), (d) $Rh_{NP}/BaLa_4Ti_4O_{15}$ (0.10 wt% Rh), (e) $Rh_{NP}@Cr_2O_3/BaLa_4Ti_4O_{15}$ (0.10 wt% Rh and 0.10 wt% Cr), and (f) $Rh_{NP}@Cr_2O_3/BaLa_4Ti_4O_{15}$ (0.75 wt% Rh and 0.31 wt% Cr). The samples (a)(b)(c) were prepared by impregnation method, whereas the samples (d)(e)(f) were prepared by photodeposition method (see section 1.3 in supporting information). In these samples, $Rh_{2-x}Cr_xO_3$(3.0 nm)/$BaLa_4Ti_4O_{15}$ including 0.10 wt% Rh and 0.15 wt% Cr (b) exhibited the highest activity.

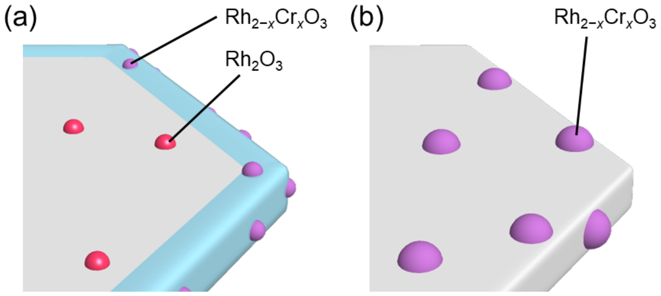

Figure S25. Schematic of (a) $Rh_{2-x}Cr_xO_3$ (1.3 nm)/$BaLa_4Ti_4O_{15}$ and (b) $Rh_{2-x}Cr_xO_3$(3.0 nm)/$BaLa_4Ti_4O_{15}$. In (a), Rh is not necessarily used for the formation of $Rh_{2-x}Cr_xO_3$ but also used for the formation of Rh_2O_3 at the flat surface of $BaLa_4Ti_4O_{15}$. Since the Rh_2O_3 particles without including Cr cannot suppress the O_2-photoreduction reaction, those particles have low activity for the water-splitting reaction (Figure S24(a)) and thereby the use of Rh for the formation of those particles leads to the decrease of the activity. In (a), there are also the excess loading of Cr_2O_3 layer, which might cause the decrease of absorption of light and thereby the decrease of the activity. On the basis of these results, it can be considered that $Rh_{2-x}Cr_xO_3$(1.3 nm)/$BaLa_4Ti_4O_{15}$ showed higher water-splitting activity than $Rh_{2-x}Cr_xO_3$(3.0 nm)/$BaLa_4Ti_4O_{15}$, because positive effects caused by miniaturization of $Rh_{2-x}Cr_xO_3$ cocatalysts (Figure 6C(b)) and selective formation of $Rh_{2-x}Cr_xO_3$ cocatalysts at the edge of $BaLa_4Ti_4O_{15}$ (Figure 2, S3, S4, and S25(a)) overcomes the negative effects caused by the formation of Rh_2O_3 particles without including Cr and the excess loading of Cr_2O_3 layer on the $BaLa_4Ti_4O_{15}$ (Figure S25(a)).

5. References

[1] K. Iizuka, T. Wato, Y. Miseki, K. Saito, A. Kudo, *J. Am. Chem. Soc.* **2011**, *133*, 20863−20868.

[2] Y. Miseki, H. Kato, A. Kudo, *Energy Environ. Sci.* **2009**, *2,* 306−314.

[3] Y. C. Zhang, R. Kershaw, K. Dwight, A. Wold, *J. Less-Common Met.* **1988**, *142*, 155−161.

[4] Y. Shichibu, Y. Negishi, T. Tsukuda, T. Teranishi, *J. Am. Chem. Soc.* **2005**, *127*, 13464−13465.

[5] H. Qian, W. T. Eckenhoff, M. E. Bier, T. Pintauer, R. Jin, *Inorg. Chem.* **2011**, *50*, 10735−10739.

[6] W. Kurashige, R. Kumazawa, D. Ishii, R. Hayashi, Y. Niihori, S. Hossain, L. V. Nair, T. Takayama, A. Iwase, S. Yamazoe, T. Tsukuda, A. Kudo, Y. Negishi, *J. Phys. Chem. C* **2018**, *122*, 13669−13681.

[7] K. Maeda, K. Teramura, D. Lu, T. Takata, N. Saito, Y. Inoue, K. Domen, *J. Phys. Chem. B* **2006**, *110*, 13753−13758.

[8] K. Maeda, K. Teramura, D. Lu, N. Saito, Y. Inoue, K. Domen, *Angew. Chem. Int. Ed.* **2006**, *45*, 7806−7809.

[9] A.L. Ankudinov, B. Ravel, J.J. Rehr, S.D. Conradson, *Phys. Rev. B*, **1998**, *58*, 7565–7576.

[10] Z.W. Seh, J. Kibsgaard, C.F. Dickens, I. Chorkendorff, J.K. Nørskov, T.F. Jaramillo, *Science*, **2017**, *355*, eaad4998.

[11] N. Kitadai, T. Yokoyama, S. Nakashima, *J. Colloid Interface Sci.* **2009**, *338*, 395−401.

[12] P. Dhamelincourt, F. J. Ramirez, *J. Raman Spec.* **1991**, *22*, 577−582.

[13] A. Barth, *Prog. Biophys. Mol. Biol.* **2000**, *74*, 141−173.
